# Oncologic Safety of Robot Thyroid Surgery for Papillary Thyroid Carcinoma: A Comparative Study of Robot versus Open Thyroid Surgery Using Inverse Probability of Treatment Weighting

**DOI:** 10.1371/journal.pone.0157345

**Published:** 2016-06-10

**Authors:** Tae-Yon Sung, Jong Ho Yoon, Minkyu Han, Yi Ho Lee, Yu-mi Lee, Dong Eun Song, Ki-Wook Chung, Won Bae Kim, Young Kee Shong, Suck Joon Hong

**Affiliations:** 1 Department of Surgery, Asan Medical Center, University of Ulsan College of Medicine, Seoul, Korea; 2 Department of Clinical Epidemiology and Biostatistics, Asan Medical Center, University of Ulsan College of Medicine, Seoul, Korea; 3 Department of Pathology, Asan Medical Center, University of Ulsan College of Medicine, Seoul, Korea; 4 Department of Internal Medicine, Asan Medical Center, University of Ulsan College of Medicine, Seoul, Korea; Kyungpook National University School of Medicine and Hospital, REPUBLIC OF KOREA

## Abstract

The aim of this study was to evaluate the oncologic safety of robot thyroid surgery compared to open thyroid surgery for papillary thyroid carcinoma (PTC). We enrolled 722 patients with PTC who underwent a total thyroidectomy with central compartment node dissection (CCND) from January 2009 to December 2010. These patients were classified into open thyroid surgery (n = 610) or robot thyroid surgery (n = 112) groups. We verified the impact of robot thyroid surgery on clinical recurrence and ablation/control-stimulated thyroglobulin (sTg) levels predictive of non-recurrence using weighted logistic regression models with inverse probability of treatment weighting (IPTW). Age, sex, thyroid weight, extent of CCND, and TNM were significantly different between the two groups (*p* < 0.05); however, there was no significant difference in recurrence between the open and robot groups (1.5% vs. 2.7%; *p* = 0.608). The proportion of patients with ablation sTg < 10.0 ng/mL and control sTg < 1.0 ng/mL was comparable between the two groups (*p* > 0.05). Logistic regression with IPTW using the propensity scores estimated by adjusting all of the parameters demonstrated that robot thyroid surgery did not influence the clinical recurrence (OR; 0.784, 95% CI; 0.150–3.403, *p* = 0.750), ablation sTg (OR; 0.950, 95% CI; 0.361–2.399, *p* = 0.914), and control sTg levels (OR; 0.498, 95% CI; 0.190–1.189, *p* = 0.130). Robot thyroid surgery is comparable to open thyroid surgery with regard to oncologic safety in PTC patients.

## Introduction

The incidence of well-differentiated thyroid carcinoma has increased worldwide, with the highest rate found in women aged 40s and early 50s [1–4]. Along with this increase, the need for better cosmetic outcomes from surgery has risen in these relatively young female patients. With this emerging trend, endocrine surgeons have focused on the postoperative quality of the scar made at the anterior neck after conventional open thyroidectomy, and tried to enhance the scar quality or develop a surgical technique to make no scar in the neck, by approaching the thyroid gland through other incision sites [5–7].

The robot-assisted gasless transaxillary thyroidectomy was developed to satisfy both the patient and surgeon by eliminating the anterior neck scar [8–15]. The results of previous reports describing the technical and/or oncologic feasibility of robot thyroid surgery compared to open thyroid surgery were comparable, and robot thyroid surgery has become increasingly popular in selected patients with papillary thyroid carcinoma (PTC) [16–19]. In a retrospective comparative study between robot and open thyroid surgery in PTC patients, we found no significant differences with regard to technical and oncologic safety [20].

Most studies have compared the short-term clinical outcomes between robot and open thyroid surgery with regard to the recurrence rate and surgical complications [14–16]. On occasion, reliable short-term prognostic factors predictive of long-term prognosis, such as serum-stimulated thyroglobulin (sTg) levels measured at the time of radioactive iodine (RAI) remnant ablation (ablation sTg) and/or 6–12 months after ablation (control sTg), were compared between the two surgical techniques [17,20–25]. In our previous study, the rate of perioperative complications was similar between robot and open thyroid surgery, and the proportion of ablation sTg levels < 10.0 ng/mL and control sTg levels < 1.0 ng/mL were comparable between the two groups (*p* > 0.05) [20]. However, these studies were susceptible to the limitations of retrospective studies such as treatment selection bias and other confounding factors. To overcome these limitations, and to gain the statistical power of a prospective study from retrospective data, various statistical methods such as propensity score (PS) matching have been adopted [25,26].

The present study evaluated the oncologic safety of robot thyroid surgery compared to open thyroid surgery in PTC patients using weighted logistic regression models with inverse probability of treatment weighting (IPTW) [27–29]. The primary endpoint of the study was clinical recurrence. Additionally, considering the short postoperative follow-up period, we adopted ablation and control sTg levels predictive of long-term non-recurrence as other endpoints.

## Materials and Methods

### Study Population

We conducted a retrospective review of 1246 patients who underwent initial thyroid surgery by a single surgeon (JHY) in Asan Medical Center (Seoul, Korea) between January 2009 and December 2010. Data were obtained from a prospectively maintained endocrine surgery database, and the study protocol was approved by Asan Medical Center institutional review board and the patient records were anonymized and deidentified prior to analysis. Among them, 1123 patients had PTC in final pathology, and 735 received total thyroidectomy with central compartment lymph node dissection (CCND). The surgical extent was based on the 2009 ATA guideline [30]. Total thyroidectomy was performed even though the tumor was less than 1.0cm size when capsular invasion, central LN metastasis or bilateral carcinoma was in suspicion. Patients with distant metastasis at the time of diagnosis and with insufficient medical records were excluded. In total, 722 patients were enrolled, and were classified into conventional open thyroid surgery (n = 610) and robot thyroid surgery groups (n = 112).

All of the patients were offered both surgical options preoperatively, and the decision of whether to perform open or robot thyroid surgery was made according to the patient’s preference. The surgical technique for robot-assisted thyroidectomy using gasless transaxillary technique has been described in detail elsewhere [8]. The clinicopathological parameters, including RAI remnant ablation status and serum ablation/control sTg levels, were compared between the two groups. After adjusting the variables for significant differences between the two groups, enrolled patients were analyzed for variable adjustment using PS and IPTW. Then, the impacts of robot surgery on clinical recurrence, ablation sTg, and control sTg levels were verified.

### Surgical Strategy

At our institution, a total thyroidectomy is performed if there are multiple or bilateral lesions and/or extrathyroid extension found during the preoperative evaluation or surgery [30]. All of the patients who were preoperatively diagnosed with PTC underwent total thyroidectomy with at least ipsilateral CCND for unilateral carcinoma; bilateral CCND was performed if lymph node (LN) enlargement in the area of the contralateral central compartment was shown on preoperative staging or surgery, and bilateral CCND was performed for bilateral carcinoma. For therapeutic and prophylactic central node dissection, CCND is performed as a routine procedure in our institution despite the preoperative cervical LN staging work-up. Therefore the patients included in this study could have underwent CCND for either prophylactic (clinical N0) reason or therapeutic (clinical N1a) reason. Therefore, we conducted our study without dividing the reasons for LN dissection. The surgical boundary of CCND includes prelaryngeal and paratracheal LNs. The thymus was not routinely removed, and mediastinal LNs were only dissected when in suspicion of pathologic LNs.

### Thyroid Remnant Ablation Protocol

Postoperative remnant RAI was performed on the indicated patients 2–3 months after the initial operation, according to the protocol established by the Endocrinology Division of Asan Medical Center [22]. Briefly, an ablative dose of 30mCi was administered to patients with a multifocal tumor and/or a tumor larger than 1.0 cm without extrathyroid extension. An ablative dose of 80 mCi was administered to patients with any tumor size less than 4.0 cm with microscopic extrathyroid extension, and an ablative dose of 150 mCi was administered to patients with a tumor size 4.0 cm or more with or without positive surgical resection margin.

At the time of remnant ablation (i.e., after thyroid hormone withdrawal or recombinant human thyroid stimulating hormone [rh-TSH] administration), serum sTg level (ablation sTg; reference range, 1.0–23.3 ng/mL) was measured with anti-Tg antibody (Ab) level (reference range, < 60.0 U/mL) when TSH level (reference range, 0.4–5.0 mU/L) was above 30.0 mU/L. A postablation whole-body scan was performed 5–7 days after administering Iodine-131.

### Postoperative Follow-up Protocol

Thyroid hormone treatment was initiated in all patients just after remnant ablation in order to decrease serum TSH to subnormal levels without inducing clinical thyrotoxicosis. Physical examinations and chest radiographies were regularly performed, and the serum sTg level was measured with anti-Tg Ab and TSH levels every 6–12 months in all patients. Diagnostic whole-body scans following thyroid hormone withdrawal or rh-TSH administration were performed every 6–12 months after remnant ablation along with the simultaneous measurement of the serum sTg level (control sTg); at that time, TSH was > 30.0 mU/L. Neck ultrasonographies were routinely performed at the same time as the diagnostic whole-body scans. When control sTg was ≥ 2.0 ng/mL and neck ultrasound (US) revealed no evidence of disease, 18F-deoxyglucose positron emission tomography or chest computed tomography (CT) were considered in order to localize persistent/remnant disease. Recurrence was defined as the appearance of pathologically proven malignant tissue and/or the appearance of metastatic lesions in the lungs, bones, and/or brain by imaging studies.

### Statistics

The parameters of the patients in both surgery groups were compared using the Student’s *t*-test for continuous variables and a Chi-square test for categorical variables. Continuous variables are presented as the mean ± standard deviation (SD) or as medians and ranges, and the categorical variables are presented in terms of percentages and absolute numbers. Multivariate analysis of all variables was done using a logistic regression model. To reduce the impact of treatment selection bias and potential confounding factors, we performed rigorous adjustments for significant differences in patient characteristics using weighted logistic regression models with IPTW. The PS was estimated by multivariate logistic regression analysis and all covariates were included to determine the PS values. With this technique, weights for patients in the robot surgery group were the inverse of (1-PS), and weights for patients in the open surgery group were the inverse of the PS. Survival outcomes were analyzed using the Kaplan-Meier method and log-rank test. All reported *p* values in this study are 2-sided, and *p* values < 0.05 were considered statistically significant. These statistical analyses were performed using R 3.1.2 software, with packages pROC and survival.

## Results

### Comparison of Clinicopathological Parameters Between Groups

The median postoperative follow-up duration was 57.2 months (range, 2.3–74.8 months). As shown in Table 1, the mean age was significantly younger in the robot thyroid surgery group than in the open thyroid surgery group (42.0 vs. 51.2 years old, *p* < 0.001). Female sex was dominant in both groups, but the proportion of female patients in the robot group was significantly higher (*p* = 0.002). The mean thyroid weight was heavier in the open group than in the robot group (24.4 vs. 18.0 g, *p* < 0.001). Ipsilateral CCND was more frequently performed in the robot group than in the open group (91.1% vs. 54.8%, *p* < 0.001).

**Table 1:** Comparison of the clinicopathological parameters between the study groups.

Parameters	Overall	Open	Robot	*p*
	n = 722	n = 610 (%)	n = 112 (%)	
Age, mean±SD (y)	49.8 ± 11.37	51.2 ± 11.27	42.0 ± 8.43	<0.001
Sex				0.002
Female	608 (84.2)	502 (82.3)	106 (94.6)	
Male	114 (15.8)	108 (17.7)	6 (5.4)	
Pathologic tumor size, mean ± SD (cm)	1.0 ± 0.64	1.0 ± 0.66	0.9 ± 0.51	0.084
Perithyroidal soft tissue invasion	454 (62.9)	381 (62.5)	73 (65.2)	0.659
Resection margin	60 (8.3)	52 (8.5)	8 (7.1)	0.764
Multiplicity	264 (36.6)	225 (36.9)	39 (34.8)	0.757
Bilaterality	172 (23.8)	144 (23.6)	28 (25.0)	0.843
Thyroiditis	205 (28.4)	170 (27.9)	35 (31.3)	0.538
Thyroid weight, mean ± SD (gm)	23.4 ± 14.37	24.4 ± 15.10	18.0 ± 7.49	<0.001
Thyroid length, mean ± SD (cm)	5.0 ± 0.88	5.0 ± 0.86	5.0 ± 0.97	0.770
Extent of central compartment node dissection				<0.001
Ipsilateral	436 (60.4)	334 (54.8)	102 (91.1)	
Bilateral	286 (39.6)	276 (45.2)	10 (8.9)	
No. of retrieved central lymph nodes, median (range)				
Ipsilateral	6 (3–10)	6 (3–10)	7 (4–9)	0.722
Bilateral	10 (6–15)	10 (6–15)	6 (2–15)	0.111
Lymph node metastasis	292 (40.4)	250 (41.0)	42 (37.5)	0.558
pT stage				0.183
pT1	259 (35.9)	218 (35.7)	41 (36.7)	
pT2	7 (1.0)	7 (1.1)	0	
pT3	417 (57.8)	348 (57.0)	69 (61.6)	
pT4a	39 (5.4)	37 (6.1)	2 (1.8)	
pN stage				0.720
pNx	16 (2.2)	14 (2.3)	2 (1.8)	
pN0	414 (57.3)	346 (56.7)	68 (60.7)	
pN1a	292 (40.4)	250 (41.0)	42 (37.5)	
TNM stage				<0.001
I	346 (47.9)	271 (43.8)	79 (70.5)	
II	1 (0.1)	1 (0.2)	0	
III	327 (45.3)	298 (48.7)	30 (26.8)	
IVA	32 (4.4)	31 (5.1)	1 (0.9)	
Recurrence	12 (1.7)	9 (1.5)	3 (2.7)	0.608
Recurrence site				
Thyroid operative bed	3 (0.4)	1 (0.2)	2 (1.8)	0.098
Lateral cervical lymph node of ipsilateral tumor site	9 (1.2)	8 (1.3)	1 (0.9)	1.000

Abbreviations: SD, standard deviation; pT, pathologic T; pN, pathologic N.

**Fig 1:**
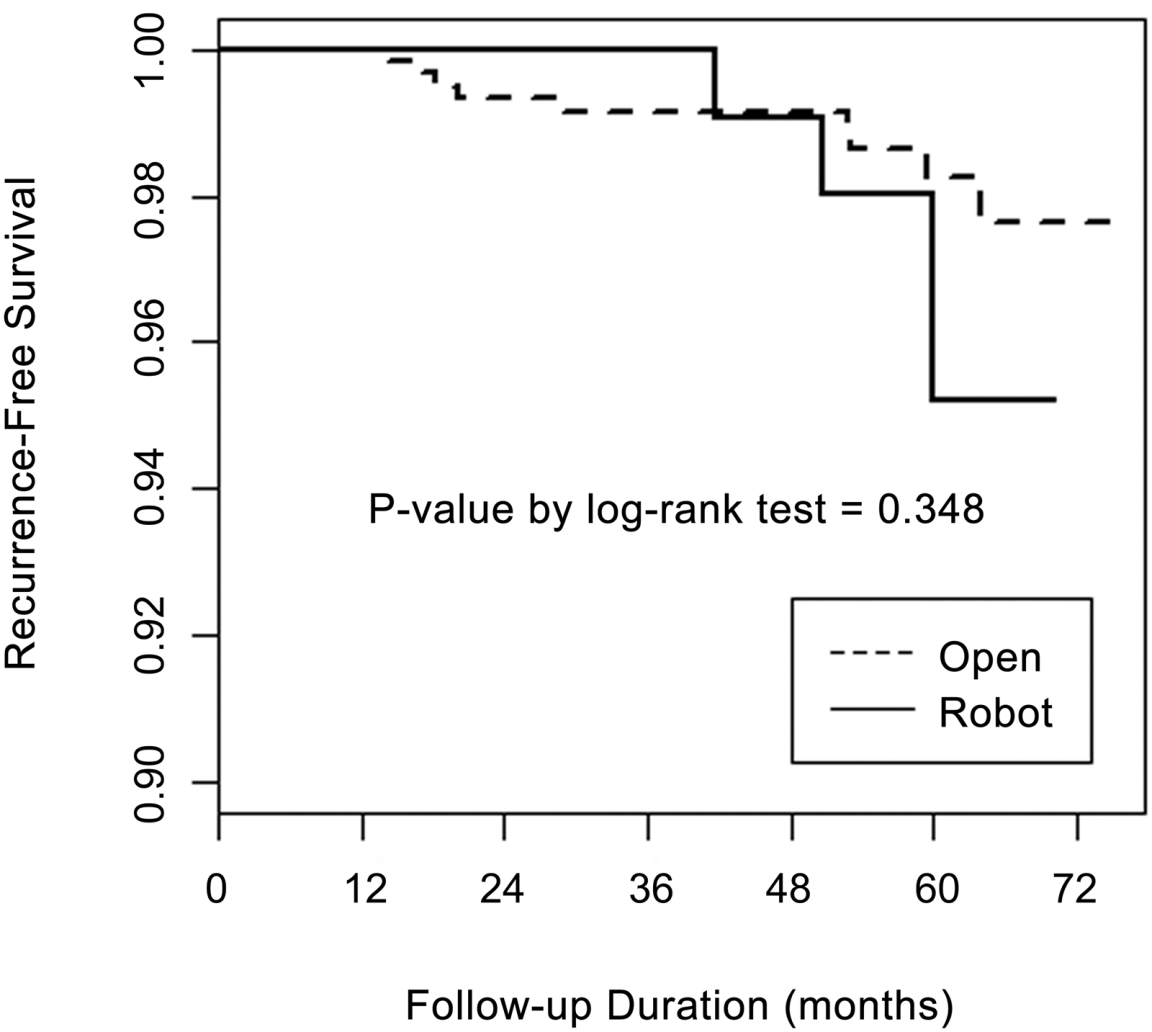
Comparison of recurrence-free survival rates between open and robot thyroid surgery using the Kaplan-Meier method.

However, the number of retrieved central LNs according to the extent of CCND was comparable between the groups (*p* > 0.05). Even though the pathological T and N stage classifications were comparable between the groups, the distribution of TNM stage [31] showed that the proportion of stage I was significantly higher in the robot than in the open group (70.5 vs. 43.8%, *p* < 0.001). Other parameters such as pathologic tumor size, perithyroidal soft tissue invasion, resection margin, multiplicity, bilaterality, thyroiditis, thyroid length and LN metastasis demonstrated no significant between-group differences (*p* > 0.05). The overall recurrence rate was 1.7% (12 of 722 patients). Nine patients in the open group (1.5%) and three in the robot group (2.7%) had recurrence showing no significant difference (*p* = 0.608 (Table 1). All recurrences were loco-regional recurrence in operative bed and lateral cervical LN area, and there was no distant metastasis during the follow-up period. As for recurrence-free survival (RFS) rates, there was no significant difference between robot and open groups (*p* = 0.348) (Fig 1).

### Comparison of Ablation and Control sTg Levels Between Groups

Postoperative RAI remnant ablation was performed in 623 patients (86.3%), including 521 patients in the open group (85.4%) and 102 patients in the robot group (91.1%), showing no significant between-group differences (*p* = 0.147) (Table 2). The dose of RAI remnant ablation applied was not different between the two groups (*p* = 0.743). Among the patients who received RAI remnant ablation, 441 patients (84.6%) in the open group and 84 patients (82.4%) in the robot group did not present anti-Tg Ab at the time of remnant ablation. The median ablation sTg (*p* = 0.300) and control sTg levels (*p* = 0.558) were comparable between the two groups, and the proportion of patients with ablation sTg < 10.0 ng/mL (*p* = 0.239) and control sTg < 1.0 ng/mL (*p* = 1.000) also did not show significant between-group differences (Table 2).

**Table 2:** Comparison of the ablation and control sTg levels between the study groups.

Parameters	Overall	Open	Robot	*p*
	n = 722 (%)	n = 610 (%)	n = 112 (%)	
RAI	623 (86.3)	521 (85.4)	102 (91.1)	0.147
RAI dose				0.743
30 mCi	275 (44.1)	228 (43.8)	47 (46.1)	
80mCi	216 (34.7)	184 (35.3)	32 (31.4)	
150 mCi	132 (21.2)	109 (20.9)	23 (22.5)	
No. of patients with a negative anti-Tg antibody level at the time of remnant ablation	525 (84.3)	441 (84.6)	84 (82.4)	0.665
Ablation sTg (ng/mL), median (range)	0.47 (0.08–61.90)	0.45 (0.08–61.90)	0.54 (0.08–47.10)	0.300
< 10.0	503 (96.3)	425 (96.4)	78 (92.9)	0.239
No. of patients with measured control sTg with a negative anti-Tg antibody level after remnant ablation	425 (84.5)	352 (82.8)	73 (93.6)	0.171
Control sTg (ng/mL), median (range)	0.08 (0.08–50.90)	0.08 (0.08–49.00)	0.08 (0.08–50.90)	0.558
< 1.0	391 (92.0)	324 (92.0)	67 (91.8)	1.000

Abbreviation: sTg, stimulated thyroglobulin; RAI, radioactive iodine; mCi, millicurie.

### Analysis of the Impact of Robot Thyroid Surgery on Clinical Recurrence, Ablation sTg and Control sTg Levels Compared to Open Thyroid Surgery with Weighted Logistic Regression

First, the propensity scores were estimated by multivariate logistic regression analysis to adjust the differences between two groups and all variables were included for adjustment as PS values. For multivariate logistic regression, the variable of surgical type (robot vs. open) was eliminated after variable selection. Therefore, we focused on the estimated decrease of the odds ratio (OR) and shortened length of the confidence interval (CI).

The OR and CI of the incidence rate (recurrence rate) of the two surgical techniques performed (robot vs. open) were OR 0.784 (95% CI 0.150–3.403) using typical IPTW compared to OR 1.367 (95% CI 0.204–5.584) for univariate logistic regression without weighting (*p* = 0.750). For cases of variable outcomes of ablation sTg level, the OR and CI of the two surgical techniques performed (robot vs. open) were OR 0.950 (95% CI 0.361–2.399) using typical IPTW compared to OR 2.123 (95% CI 0.741–5.358) for univariate logistic regression without weighting (*p* = 0.914). For cases of outcome variables of control sTg levels, the OR and CI of the two surgical techniques performed (robot vs. open) were OR 0.498 (95% CI 0.190–1.189) using typical IPTW compared to OR 0.940 (95% CI 0.309–2.354) for univariate logistic regression without weighting (*p* = 0.130). In all of the cases, the ORs were cut by 50%, presenting more realistic estimated results using IPTW (Table 3). In addition, these results show that there were no significant differences with regard to clinical recurrence and ablation/control sTg levels based on surgical type (*p* > 0.050)

**Table 3:** Impact of robot thyroid surgery on clinical recurrence, ablation sTg levels and control sTg levels compared to open thyroid surgery with weighted logistic regression.

	Odds ratio	95% CI	*p*
Clinical recurrence			
Logistic regression without weight	1.367	0.204–5.584	0.696
Logistic regression with IPTW	0.784	0.150–3.403	0.750
Ablation sTg			
Logistic regression without weight	2.123	0.741–5.358	0.129
Logistic regression with IPTW	0.950	0.361–2.399	0.914
Control sTg			
Logistic regression without weight	0.940	0.309–2.354	0.903
Logistic regression with IPTW	0.498	0.190–1.189	0.130

Abbreviations: sTg, stimulated thyroglobulin; CI, confidence interval.

## Discussion

Retrospective studies describing the oncologic safety of robot thyroid surgery have been limited due to the bias of surgical technique selection, and these limitations are from both the patient’s and surgeon’s perspectives. The patient’s perspective is that younger and/or female patients may pay more attention to the cosmetic benefits of robot thyroid surgery compared to older and/or male patients [13,14,17,20]. This preference could influence the oncologic outcomes of robot thyroid surgery, because both female gender and younger age are better prognostic factors than male gender and/or older age. The surgeon’s perspective is more complex. Robot thyroid surgery cannot be applied to all thyroid carcinoma surgeries, and institutions that perform robot surgery for thyroid carcinoma have their own inclusion criteria. In addition, the inclusion criteria could be dependent on the surgeon’s experience and ability to perform the surgery, which is usually applied to early stage thyroid carcinoma without overt extrathyroid extension, especially to posterior capsule of thyroid gland, and the cases which do not necessitate the therapeutic or prophylactic level VII LN dissection since this is difficult to achieve mechanically by robot surgical techniques. Therefore, the robot group might have involved patients in the earlier TNM stage [13], who underwent less extended LN dissection. Furthermore, patient’s anthropometric perspectives like the length of neck, volume or height of thyroid gland, and location of major vessels around the thyroid gland might also influence the surgeon’s selection of either robot or open thyroid surgery unintentionally. These various confounding factors in retrospective studies might cause treatment selection bias and make it difficult to accurately compare the oncologic outcomes between robot and open thyroid surgery.

The present study also demonstrated the problems mentioned above. The patients in the robot group were significantly younger and female dominant. The mean thyroid weight was significantly heavier in the open group compared to the robot group. Furthermore, in the robot group compared to open group, ipsilateral CCND was more frequently performed compared to bilateral CCND. However, the number of retrieved central LNs according to the extent of CCND, either ipsilateral or bilateral, was comparable between the groups. The distribution of TNM stage showed a higher proportion of stage I in the robot group, as aforementioned. The postoperative complications related to two different surgical techniques were described in our previous study published in 2013 that there was no significant difference in overall complication rates and the results were similar in present study.

One limitation of this study was a relatively short follow-up period for accurately evaluating the impact of robot thyroid surgery on clinical recurrence. Therefore, we used ablation and control sTg levels as other endpoints. The ablation sTg and control sTg levels predict recurrence and were reliable short-term prognostic factors for patients who underwent total thyroidectomy and RAI remnant ablation [21,23,24,32,33]. One study reported that the negative predictive value (NPV) for recurrence using an ablation sTg level < 10.0 ng/mL was 94.2%, at least among patients without biochemical or structural evidence of disease at the initial surveillance or subsequent follow-up examinations [33]. Han et al [23] reported that only about 1% of patients with a control sTg level < 1.0 ng/mL developed clinical recurrence.

Recently, to overcome the limitation related to the bias of retrospective studies, various statistical methods such as PS matching have been adopted to gain the statistical power of prospective study [25]. PS matching method is basically used for an equally large sample case. In that case, there is rarely a loss of information after matching. However, for small samples or unequally divided sample cases, another method has been recommended instead of PS matching since there are significant losses of information. Based on this concept, we analyzed our data using weighted logistic regression models with IPTW, and evaluated the impact of robot thyroid surgery on clinical recurrence, ablation sTg and control sTg levels compared to open surgery to predict the long-term prognosis. Logistic regression with IPTW showed that there was no significant difference between the open and robot groups with regard to clinical recurrence, ablation sTg and control sTg levels.

To the best of our knowledge, this study was the first to compare the oncologic safety between robot and open thyroid surgery for PTC using the IPTW method, demonstrating no significant between-group difference with regard to recurrence. In conclusion, we believe that the oncologic safety of robot thyroid surgery is comparable to open thyroid surgery and is applicable to the surgical treatment of PTC patients.
